# Anemia Risk Prediction Model for Osteosarcoma Patients Post‐Chemotherapy Using Artificial Intelligence

**DOI:** 10.1002/cam4.70427

**Published:** 2024-12-02

**Authors:** Zhiping Su, Zhiwei Nong, Feihong Huang, Chengxing Zhou, Chaojie Yu

**Affiliations:** ^1^ Department of Bone and Soft Tissue Surgery Guangxi Medical University Cancer Hospital Nanning Guangxi Zhuang Autonomous Region China; ^2^ Guangxi Medical University Nanning Guangxi Zhuang Autonomous Region China; ^3^ Department of Radiation Oncology Guangxi Medical University Cancer Hospital Nanning Guangxi Zhuang Autonomous Region China; ^4^ Department of Ultrasound The People's Hospital of Guangxi Zhuang Nanning China; ^5^ Guangxi Key Laboratory of Regenerative Medicine, Orthopaedic Department The First Affiliated Hospital of Guangxi Medical University Nanning Guangxi Zhuang Autonomous Region China

**Keywords:** anemia, artificial intelligence, chemotherapy, diagnostic model, osteosarcoma

## Abstract

**Objective:**

This study aimed to develop a machine learning model for predicting anemia post‐chemotherapy in osteosarcoma patients.

**Methods:**

Clinical data from 631 osteosarcoma patients were collected, and after data filtering, a training set and validation set were created. Various statistical tests were conducted on the data, and single‐factor and multiple‐factor logistic regression analysis, random forest (RF), support vector machine (SVM), and least absolute shrinkage and selection operator (LASSO) were used to construct risk prediction models. A new model was created by intersecting the above models to identify common risk factors, and a nomogram was developed to display the new model. The model's performance was validated using the validation set.

**Results:**

Twenty‐five risk factors were identified in the anemia group compared to the non‐anemia group (*p* < 0.05). Single‐factor logistic regression analysis identified 22 risk factors (AUC 0.895), whereas multiple‐factor logistic regression analysis identified 8 risk factors (AUC 0.872), RF identified 7 risk factors (AUC 0.851), SVM identified 16 risk factors (AUC 0.851), and LASSO identified 19 risk factors (AUC 0.902). Five common risk factors (ALB, Ca, CREA, D‐dimer, and ESR) were identified through model intersection, yielding a new model with an AUC of 0.85. Internal validation of the new model showed an AUC of 0.802, indicating high predictive ability. A web model application was created (https://anemic‐prediction‐of‐osteosarcoma.shinyapps.io/DynNomapp/).

**Conclusion:**

The developed risk prediction model based on clinical and laboratory data can aid in individualized diagnosis and treatment of anemia in osteosarcoma patients post‐chemotherapy.

**Abbreviations**
AFP: alpha fetoprotein
ALB: albumin
ALP: alkaline phosphatase
ALT: alanine transaminase
ALT: glutamic‐pyruvic transaminase
APTT: activated partial thromboplastin time
AST: aspartate ami‐notransferase
AUC: area under the curve
BASOR: basophilic granulocyte
BASOR%: percentage of basophilic granulocyte
BLT: blood platelet
B‐lym: B lymphocytes
BMl: body mass lndex
C3: complement C3
C4: complement C4
Ca: serum calciumion
CA‐I25: carbohydrate antigen‐I25
CA‐I53: carbohydrate antigen‐I53
CA‐I99: carbohydrate antigen‐I99
Ccr: creatinine clearance rate
CEA: carcinoembryonic antigen
CIC: clinical impact curve
C‐index: the concordance index
CK: creatine kinase
CK‐MB: creatine kinase isoenzyme
Cl: serum chlorideion
CREA: creatinine
CRP: C‐reactive protein
cyfra2I‐I: human CYFRA2I‐I antigen
DCA: calibration curve, decision curve analysis
EOSR: eosinophils
EOSR%: percentage of eosinophils
ESR: erythrocyte sedimentation rate
FBG: fasting blood glucose
FlB: ibrinogen
FT3: free triiodothyronine
FT4: free thyroxine
GGT: Y‐glutamyl transpeptadase
Han: the Han nationality
Han: the Han nationality
Hb: hemoglobin
HDL‐C: high‐density lipoprotein
hs‐CRP: high‐sensitivity C‐reactive protein
hs‐Tnl: hypersensitive plasma troponin l
K: serum potassiumion
LASSO: least absolute shrinkage and selection operator
LDH: lactic dehydrogenase
LDL‐C: low‐density lipoprotein
lgA: immunoglobulin A
lgG: immunoglobulin G
lgM: immunoglobulin M
LPS: lipase
LYM: lymphocytes
LYM%: percentage of lymphocytes
Miao: the Miao nationality
MO: monocyte
MO%: monocyte percentage
Mulao: the Mulao nationality
MYO: myohemoglobin
NA: serum sodiumion
NEU: neutrophilic gran‐ulocyte
NEU%: neutrophilic granulocyte percentage
NKC: natural lxiller cell
PCT: procalcitonin
PT: prothrombin time
RBC: red blood cell
RF: random forest
ROC: receiver operating characteristic curve
SCC Ag: squamous cell carcinoma antigen
SF: serum ferritin
SVM: support vector machine
T3: triiodothyronine
T4: thyroxine
TAG: triglyceride
TCHO: total cholesterol
TG: triglyceride
Th‐lym: helper T lymphocytes
T‐lym: cytotoxic T lymphocytes
TP: total protein
TRF: transferrin
TSH: thyroid stimulating hormone
Ts‐lym: suppressor T cell
TT: thrombin time
UA: uric acid
UREA: ureophil
WBC: white blood cell
Yao: the Yao nationality
Zhuang: the zhuang nationality

## 1 Introduction

Osteosarcoma, comprising 35% of primary bone tumors, presents as the most common malignant bone tumor, predominantly affecting children and adolescents in the limbs [1, 2]. Despite advancements, its prognosis remains poor, with an increasing incidence rate of 0.3% annually over the past decade [3]. Adjuvant chemotherapy, while improving the 5‐year survival rate, poses risks such as myelosuppression‐related anemia [4]. Early detection of anemia is crucial for tailored treatment and patient prognosis, yet current methods rely on hemoglobin levels, limiting timely intervention [5].

Nomograms offer a simplified visual representation of complex statistical models, aiding clinicians in predicting clinical events with minimal learning costs [6, 7, 8]. Concurrently, the integration of artificial intelligence (AI) in medicine, including oncology, genomics, and medical imaging, presents a promising frontier [9, 10, 11]. Machine learning, capable of handling vast and diverse medical data, intersects with healthcare, fueling research in recent years [12].

Currently, no studies have utilized AI in predicting chemotherapy‐induced anemia in osteosarcoma patients. We aim to leverage machine learning techniques to identify optimal risk factor combinations from patient clinical and laboratory data, constructing a predictive model for anemia. Early detection through this model can significantly enhance patient prognosis, offering crucial decision support for personalized clinical management.

## 2 Materials and Methods

### 2.1 Research Object

We retrospectively collected data from 631 osteosarcoma patients admitted to our hospital between January 2013 and June 2021. Inclusion criteria required biopsy‐confirmed osteosarcoma, chemotherapy treatment, complete hematological test results, standard treatment, and full follow‐up data. Exclusion criteria comprised patients without chemotherapy, presence of other tumors, incomplete laboratory data, or lost follow‐up [13]. After excluding 314 cases, 317 eligible patients were included, of which 171 had anemia (hemoglobin < 120 g/L) and 146 did not. Patient data were de‐identified, and they were randomly divided into a training set (*n* = 317) and a verification set (*n* = 93) with a 7:3 ratio. This retrospective study received approval from the Medical Ethics Committee of the Affiliated Cancer Hospital of Guangxi Medical University, adhering to the Declaration of Helsinki. Informed consent was waived due to the study's retrospective nature.

### 2.2 Data Collection and Analysis

In this study, we collected 24 clinical parameters and 76 laboratory parameters from patients with osteosarcoma. Clinical parameters included demographic information (gender, age, marital status, nationality), anthropometric measures (blood pressure, height, weight, BMI), medical history (smoking, drinking, complications, surgery details, primary tumor site, TNM stage, Ki‐67 expression), and treatment methods (chemotherapy, radiotherapy, targeted therapy, immunotherapy). Laboratory parameters encompassed various hematological and biochemical tests, such as blood type, WBC, LYM%, MO%, NEU%, LYM, MO, NEU, RBC, Hb, BLT, EOSR%, BASOR%, EOSR, BASOR, ALT, TP, ALB, TRF, WBC, LYM, Mo%, NEU%, LYM, Mo, NeU, RBC, HB, BLT, EOSR%, BASor%, AST, AST/ALT, GGT, ALP, LDH, LPS, SF, K, NA, Cl, Ca, UREA, CREA, Ccr, UA, CK, CK‐MB, FBG, hs‐TnI, MYO, PT, APTT, FIB, TT, D‐dimer, CRP, hs‐CRP, ESR, PCT, C3, C4, IgG, IgA, IgM, T‐lym, Th‐lym, Ts‐lym, Th/Ts, NKC, B‐lym, CEA, AFP, CA‐125, CA‐153, CA‐199, SCC‐Ag, cyfra21‐1, TSH, T4, T3, FT4, FT3, TG, TCHO, TAG, HDL‐C, and LDL‐C. All of our patients have received standard chemotherapy regimens for osteosarcoma and have not been treated with any additional drugs outside this regimen that could potentially cause anemia in patients with osteosarcoma.

### 2.3 Method of Model Construction

#### 2.3.1 General Statistical Analysis

The clinical data of the two groups were divided into anemia group and non‐anemia group. The measurement data were tested for normality. Those with normal distribution were represented by *x* ± *s*. Independent sample *t* test was used for comparison between groups. Those who did not conform to the normal distribution were represented by M (Q1, Q3), and Mann–Whitney *U* test was used for comparison between groups. Counting data were expressed in proportion and Chi‐square test was used for comparison between groups. Statistical tests were all bilateral, and *p* < 0.05 was considered to be statistically significant.

#### 2.3.2 Correlation Analysis

After general statistical analysis, indicators with *p* < 0.05 and statistical significance will be obtained for correlation analysis, and the correlation coefficient can be used to evaluate the degree of correlation among indicators.

#### 2.3.3 Single Factor Analysis Method

We used SPSS 25.0 software to conduct one‐way logistic regression analysis on 24 clinical variables and 76 laboratory parameters, following *t*‐tests, *U* tests, or Chi‐square tests. The statistically significant risk factors (*p* < 0.05) identified were used to formulate the first prediction model for anemia following chemotherapy in osteosarcoma patients.

#### 2.3.4 Multi‐Factor Analysis Method

Similarly, SPSS 25.0 was used to perform *t*‐tests, *U* tests, or Chi‐square tests on the dataset, followed by multi‐factor logistic regression analysis to ascertain the interplay between variables. The significant risk factors (*p* < 0.05) identified were utilized to establish the second prediction model.

#### 2.3.5 Random Forest Method

Random Forest, an ensemble learning algorithm, was applied to build the third prediction model. This method combines multiple decision trees, each using randomly selected features, to create an integrated classifier. Its inherent randomness and bootstrapping convergence contribute to robustness and accuracy [14]. We used the risk factors identified from initial tests to construct this model.

#### 2.3.6 Support Vector Machine (SVM) Method

Using the principles of VC dimension theory and structural risk minimization, SVM was used to construct the fourth prediction model. This supervised learning algorithm optimizes the classification boundary to achieve maximal generalization ability and classification accuracy [15]. Risk factors identified from initial tests were input into the SVM algorithm for model construction.

#### 2.3.7 Least Absolute Shrinkage and Selection Operator (LASSO) Method

LASSO, a regularization technique, was applied to construct the fifth prediction model. By penalizing regression coefficients and promoting sparsity, LASSO effectively handles collinearity and generates a refined model [16, 17]. We used risk factors identified from initial tests as input for the LASSO method.

#### 2.3.8 Intersection Model

Lastly, we combined the prediction models generated by the aforementioned methods to identify the optimal combination of risk factors. By integrating insights from multiple approaches, we constructed the sixth prediction model to enhance predictive accuracy and robustness.

### 2.4 Model Evaluation Methods

**FIGURE 1:**
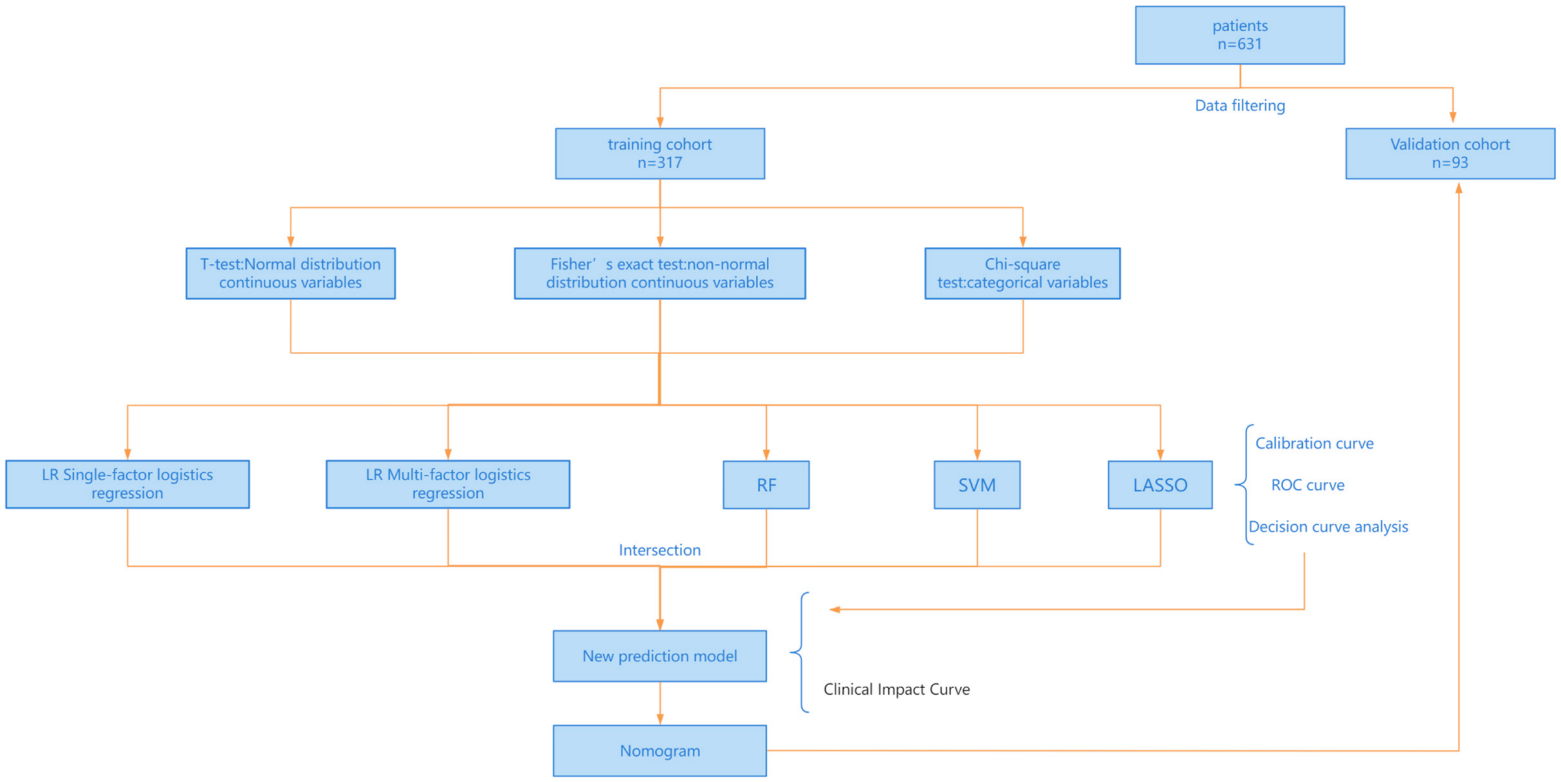
Flowchart depicting the development and evaluation process of the anemia risk prediction model following chemotherapy for osteosarcoma.

Following the development of the anemia prediction model using statistical analysis and machine learning techniques, its performance was assessed using several metrics. The concordance index (C‐index), receiver operating characteristic (ROC) curve, area under the curve (AUC), calibration curve, decision curve analysis (DCA), and clinical impact curve (CIC) were used to validate the reliability and efficacy of these models. An AUC value greater than 0.5 indicates favorable predictive capability, with higher values signifying stronger predictive performance. Figure 1 illustrates the data processing workflow for osteosarcoma patients, along with the establishment and validation of the anemia risk prediction model following chemotherapy.

## 3 Results

### 3.1 Baseline Characteristics of Patients

Table 1 presents the demographic and clinical characteristics of osteosarcoma patients included in this study. Following rigorous screening, 317 eligible patients were enrolled, with internal verification performed to assess model performance. The patient cohort was randomly divided into training and verification sets, maintaining a ratio of ~7:3. Among the training set, consisting of 317 patients, 146 were classified into the anemia group and 171 into the non‐anemia group. Similarly, the verification set comprised 48 anemia cases and 45 non‐anemia cases.

**TABLE 1:** Baseline data of patients with osteosarcoma.

Variables	Training cohort			Validation cohort		
	Anemia (*n* = 146)	Non‐anemia (*n* = 171)	*p*	Anemia (*n* = 48)	Non‐anemia (*n* = 45)	*p*
Gender			0.0038*			0.941
Male	73	114		27	24	
Femle	73	57		21	21	
Marriage			0.5926			0.826
Unmarried	87	108		33	29	
Married	59	63		15	16	
Nation			0.5358			0.335
Han	103	112		35	29	
Zhuang	39	57		11	16	
Yao	2	1		1	0	
Miao	1	1		1	0	
Mulao	1	0				
Smoke			0.1122			0.941
No	138	152		46	42	
Yes	8	19		2	3	
Drink			0.0705			0.955
No	143	159		47	43	
Yes	3	12		1	2	
Complication			0.9062			0.773
No	63	76		21	22	
Yes	83	95		27	23	
Surgery			0.0485*			0.023*
No	36	26		11	2	
Yes	110	145		37	43	
Type of surgery						
Amputation	26	23		5	4	
Limb salvage	84	122		32	39	
Primary site			1			1
Axial skeleton	30	35		11	10	
Limb bone	116	136		37	35	
M stage			0.5274			1
No	75	95		30	28	
Yes	71	76		18	17	
M stage			0.6751			0.929
No	115	139		37	36	
Yes	31	32		11	9	
Clinical stages (AJcc 8th)			0.492			1
I–II	66	85		25	23	
III–IV	80	86		23	22	
Blood types			0.1326			0.194
O	77	74		25	23	
A	25	39		8	10	
B	40	46		15	9	
AB	4	12		0	3	
Tumor size (mm)	10.217	9.214	0.0674	9.054	9.978	0.3313
Systolic pressure (mmHg)	113.678	111.830	0.261	110.812	112.067	0.6964
Diastolic pressure (mmHg)	73.932	72.322	0.2117	72.271	72.644	0.8631
BMI (kg/m^2^)	18.677	29.487	0.3011	157.667	160.378	0.334
Age	26.829	25.439	0.4351	46.979	54.422	0.0352
Blood routine						
WBC (×10^9^/L)	7.458	7.530	0.8069	7.530	7.935	0.5007
LYM%	26.520	29.358	0.0171*	24.956	30.059	0.0152*
MO%	7.643	6.721	0.0014*	7.636	6.706	0.0438*
NEU%	62.059	59.544	0.0594	64.398	58.101	0.011*
LYM (×10^9^/L)	1.812	2.091	0.0003*	1.711	2.193	0.0001*
MO (×10^9^/L)	0.546	0.503	0.0963	0.559	0.530	0.5892
NEU (×10^9^/L)	4.860	4.624	0.3692	5.038	4.882	0.7734
BLT (×10^9^/L)	348.449	303.907	0.0003*	365.103	321.482	0.0393*
EOSR%	2.769	3.522	0.0849	2.331	3.278	0.2231
BASOR%	0.528	0.521	0.8644	0.533	0.604	0.485
EOSR (×10^9^/L)	0.196	0.272	0.0961	0.162	0.285	0.245
BASOR (×10^9^/L)	0.036	0.038	0.619	0.036	0.044	0.2901
Liver function						
ALT (U/L)	20.452	20.706	0.9299	23.646	25.835	0.7907
TP (g/L)	66.623	70.277	< 0.0001*	68.206	70.858	0.0275*
ALB (g/L)	36.793	42.678	0.0001*	37.908	41.499	0.0008*
TRF (g/L)	3.124	2.521	0.5091	2.160	2.543	0.0001*
AST (U/L)	27.692	24.733	0.0793	26.667	26.782	0.9735
AST/ALT	1.861	1.708	0.1949	1.637	1.578	0.7416
GGT (U/L)	35.137	31.258	0.3767	35.729	37.068	0.8881
ALP (U/L)	423.041	357.291	0.5089	333.625	402.946	0.6689
LDH (U/L)	358.877	290.662	0.361	264.208	247.380	0.5679
LPS (U/L)	31.105	31.597	0.8917	37.820	28.786	0.4165
SF (μg/L)	269.639	207.765	0.0205*	258.505	202.050	0.16
Electrolyte						
K (mmol/L)	4.168	4.291	0.0068*	4.213	4.249	0.626
Na (mmol/L)	139.466	142.175	0.1239	139.604	140.333	0.1679
Cl (mmol/L)	99.651	100.606	0.3145	100.042	98.704	0.5471
Ca (mmol/L)	2.256	2.365	< 0.0001*	2.269	2.337	0.0234*
Renal function						
UREA (mmol/L)	5.619	4.601	0.4895	3.782	4.400	0.0174*
CREA (μmol/L)	54.615	64.201	< 0.0001*	51.417	61.818	0.0047*
Ccr (mL/min)	99.188	95.544	0.1681	105.627	98.926	0.1609
UA (μmol/L)	293.818	333.907	0.0003*	287.113	331.579	0.0468*
Cardiovascular function						
CK (U/L)	104.174	97.333	0.7163	59.489	80.212	0.0147*
CK‐MB (U/L)	15.268	13.742	0.3735	11.363	14.395	0.0187*
FBG (mmol/L)	4.635	4.615	0.8415	4.684	4.577	0.5156
hs‐TnI (pg/mL)	3.364	3.615	0.548	3.275	3.023	0.4249
MYO (ng/mL)	90.679	87.893	0.8971	85.443	96.422	0.8076
Coagulation function						
PT (Sec)	12.720	12.282	0.0019*	12.463	12.088	0.0887
APTT (Sec)	32.227	30.136	0.0003*	31.844	29.784	0.0434*
FIB (g/L)	4.231	3.646	0.0001*	4.267	3.698	0.0375*
TT (Sec)	16.078	17.094	0.1416	15.738	16.238	0.1473
D‐dimer (μg/mL)	3.678	1.365	< 0.0001*	3.910	1.222	0.0166*
Inlammatory factors						
CRP (mg/L)	29.849	14.526	< 0.0001*	28.406	13.213	0.0213*
hs‐CRP (mg/L)	12.760	7.217	0.0216	15.365	8.345	0.2322
ESR (mm/h)	49.626	31.210	< 0.0001*	51.007	34.238	0.0014*
PCT (ng/mL)	2.664	1.228	0.0445*	1.448	1.162	0.15
Humoral immune factors						
C3 (g/L)	1.214	1.170	0.1129	1.270	1.160	0.0435*
C4 (g/L)	0.303	0.295	0.4799	0.305	0.296	0.5706
IgG (g/L)	12.869	13.131	0.7333	12.829	15.497	0.2811
IgA (g/L)	2.500	2.281	0.0158*	2.605	2.313	0.0716
IgM (g/L)	1.292	1.319	0.629	1.213	1.375	0.1177
Cellular immune factors						
T‐lym (%)	70.070	69.255	0.3953	69.840	70.079	0.8917
Th‐lym (%)	62.932	38.085	0.2487	109.303	37.888	0.3312
Ts‐lym (%)	25.309	25.465	0.8334	25.191	26.617	0.3446
Th/Ts	1.621	1.529	0.164	1.630	1.500	0.3077
NKC(%)	9.898	10.605	0.3126	10.417	10.327	0.9441
B‐lym(%)	12.935	12.896	0.9526	12.370	11.809	0.6568
Adenocarcinoma markers						
CEA (ng/mL)	1.230	1.437	0.0078*	1.140	1.462	0.0277*
AFP (ng/mL)	1.977	2.205	0.183	1.733	2.493	0.0749
CA‐125 (U/mL)	26.688	17.437	0.0552	17.887	14.732	0.172
CA‐153 (U/mL)	12.981	11.461	0.042*	12.138	11.880	0.8245
CA‐199 (U/mL)	12.329	15.392	0.3632	14.313	13.581	0.8189
Squamous carcinoma markers						
SCC Ag (ng/mL)	0.804	0.865	0.1503	0.766	0.913	0.0271*
cyfra2I‐I (ng/mL)	1.904	2.068	0.4608	2.115	1.762	0.3681
Thyroid function						
TSH (μIU/mL)	2.062	2.022	0.8325	1.864	2.210	0.2545
T4 (nmol/L)	93.476	95.800	0.1663	93.117	97.309	0.2306
T3 (nmol/L)	1.519	1.561	0.1669	1.553	1.567	0.7852
FT4 (pmol/L)	13.120	13.321	0.1393	13.272	13.520	0.2651
FT3 (pmol/L)	4.499	4.634	0.0543	4.639	4.672	0.7167
Serum lipid						
TG (mmol/L)	11.223	12.887	0.2594	11.240	10.527	0.4266
TCHO (mmol/L)	4.274	4.416	0.0542	4.192	4.682	0.0008*
TAG (mmol/L)	1.104	1.117	0.7524	1.119	1.235	0.1572
HDL‐C (mmol/L)	1.137	1.165	0.2123	1.135	1.142	0.8756
LDL‐C (mmol/L)	2.772	2.862	0.1459	2.688	3.084	0.0016*

*Note:* Variables with a significance level of *p* < 0.05 in the table have been denoted with an asterisk (*).

Of the total patients, 187 were male and 130 were female, with ages ranging from 4 to 75 years and a mean age of 26.1 years. Upon laboratory examination, patients were stratified into anemia and non‐anemia groups based on established diagnostic criteria. Notably, statistical analysis (*t*‐test, Mann–Whitney *U* test, or *χ* ^2^ test) of 100 included parameters revealed that Gender, Surgery Type, LYM%, MO%, LYM, BLT, TP, ALB, SF, K, CA, CREA, UA, PT, APTT, FIB, D‐dimer, CRP, hs‐CRP, ESR, PCT, IgA, CEA, and CA‐153 exhibited significant associations (*p* < 0.05), as detailed in Table 1.

### 3.2 Correlation Analysis

**FIGURE 2:**
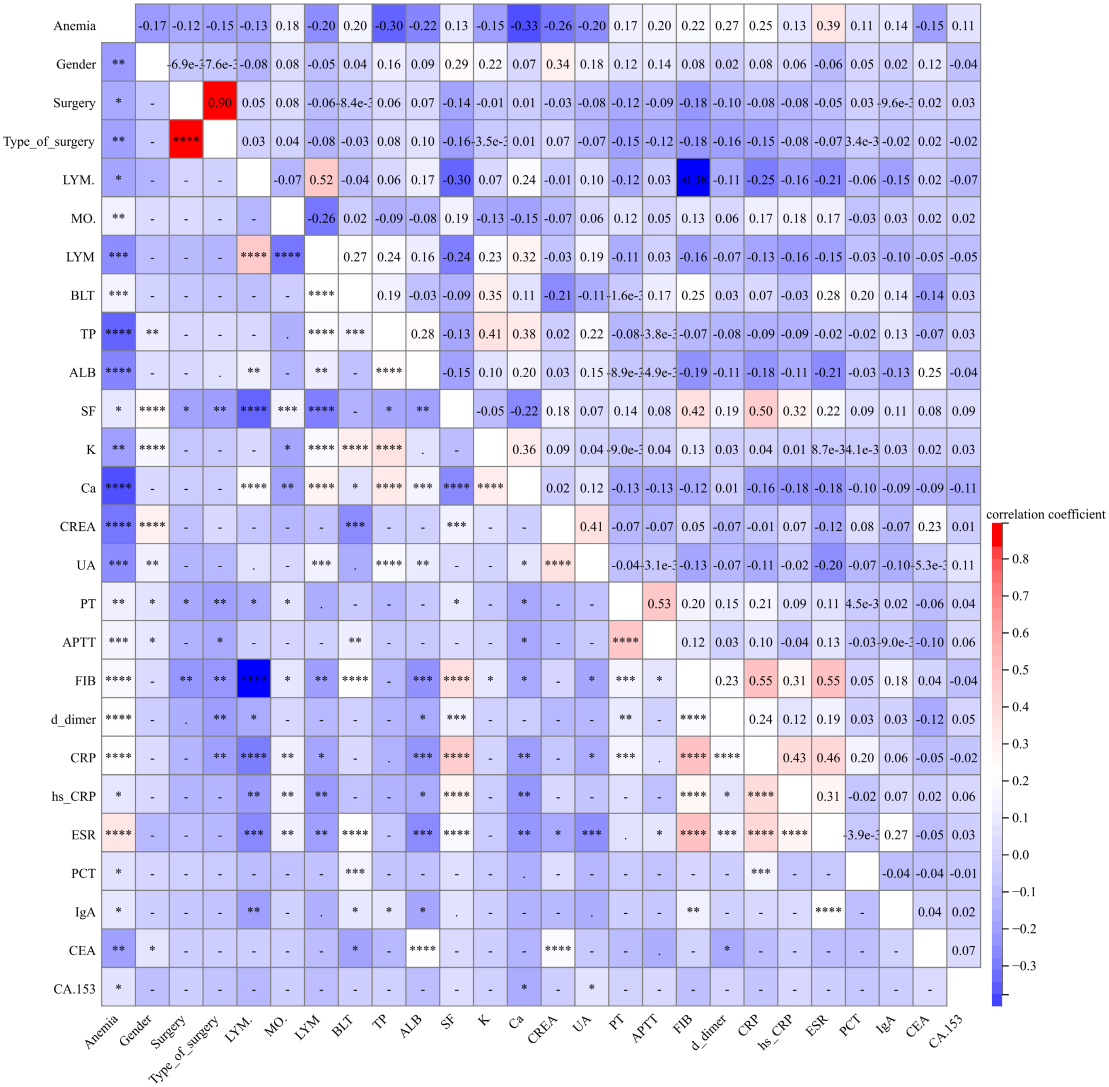
Correlation analysis of risk factors for anemia following chemotherapy in osteosarcoma patients. (Comprising a total of 317 patients (130 females and 187 males) aged between 10 and 73 years.)

Following the *t*‐test, correlation analysis was conducted on 25 identified independent risk factors. Results revealed significant correlations among certain variables. Specifically, surgery type exhibited a strong correlation (Pearson correlation coefficient: 0.90) with the performance of surgery. Additionally, LYM displayed a moderate correlation (Pearson correlation coefficient: 0.52) with LYM%, whereas SF exhibited a moderate correlation (Pearson correlation coefficient: 0.50) with CRP. Furthermore, PT demonstrated a moderate correlation (Pearson correlation coefficient: 0.53) with APTT, and FIB displayed moderate correlations (Pearson correlation coefficient: 0.55) with both CRP and ESR (Figure 2). These findings suggest the potential for reducing the inclusion of redundant risk factors in each model, thereby minimizing machine learning processing times and streamlining clinician analytical metrics.

### 3.3 Construction of Model

#### 3.3.1 Construction of a Single‐Factor Logistic Model

**FIGURE 3:**
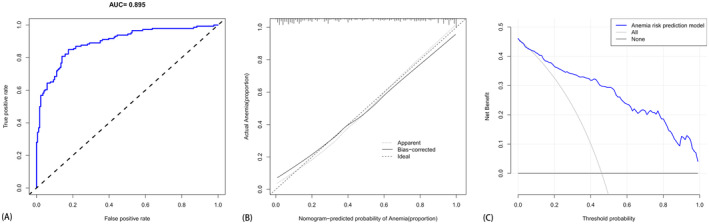
(A) The ROC curve for anemia prediction based on single‐factor logistic regression analysis, whereas (B) illustrates the calibration curve and (C) presents the clinical decision analysis curve for anemia prediction using single‐factor logistic regression analysis. (Comprising a total of 317 patients (130 females and 187 males) aged between 10 and 73 years.)

The outcomes of single‐factor logistic regression analysis revealed statistically significant differences across 22 indices, encompassing gender, surgical intervention, LYM%, MO%, LymphoCount, PLT, TP, ALB, SF, K, Ca, CREA, UA, PT, APTT, FIB, D‐dimer, CRP, ESR, IgA, CEA, and CA‐153. These indices were identified as candidate variables for predicting post‐chemotherapy anemia in osteosarcoma patients (*p* < 0.05), as delineated in Table 2. Subsequently, these 22 indicators were used in single‐factor logistic regression to construct the anemia risk prediction model 1. Model 1 yielded a predictive area under the curve (AUC) of 0.895 (see Figure 3A), indicating robust discrimination capability. The calibration curve (Figure 3B) demonstrated the model's accuracy in estimating the risk probability. Furthermore, the clinical DAC (Figure 3C) indicated the potential benefits to patients and the practical utility of this model.

**TABLE 2:** Results of single‐factor logistic regression analysis.

Variables	Univariate analysis		
	OR	95% CI	*p*
Gender			
Female	Reference		
Male	0.500	0.317–0.787	0.003
Surgery			
No	Reference		
Yes	0.548	0.312–0.961	0.036
LYM%	0.974	0.953–0.996	0.019
MO%	1.168	1.056–1.291	0.002
LYM%	0.528	0.372–0.751	0.000
BLT	1.004	1.002–1.006	0.000
TP	0.897	0.860–0.935	0.000
ALB	0.779	0.729–0.833	0.000
SF	1.001	1.000–1.002	0.026
K	0.459	0.259–0.813	0.008
Ca	0.010	0.002–0.052	0.000
CREA	0.969	0.955–0.982	0.000
UA	0.996	0.993–0.998	0.000
PT	1.335	1.108–1.608	0.002
APTT	1.085	1.037–1.136	0.000
FIB	1.431	1.192–1.717	0.000
D‐dimer	1.216	1.112–1.330	0.000
CRP	1.021	1.011–1.031	0.000
ESR	1.042	1.028–1.056	0.000
IgA	1.424	1.059–1.916	0.019
CEA	0.620	0.430–0.893	0.010
CA‐153	1.037	1.000–1.076	0.049

#### 3.3.2 Multi‐Factor Logistic Regression Analysis Model

**FIGURE 4:**
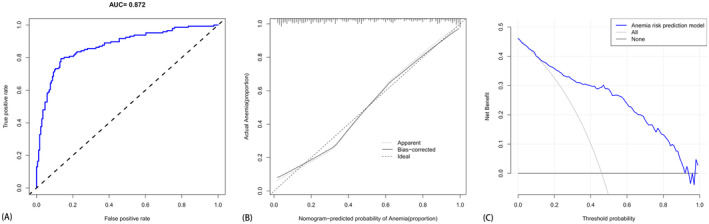
(A) The ROC curve for anemia prediction based on multi‐factor logistic regression analysis, whereas (B) presents the calibration curve, and (C) depicts the clinical decision analysis curve for anemia prediction using multi‐factor logistic regression analysis. (Comprising a total of 317 patients (130 females and 187 males) aged between 10 and 73 years.)

The findings from the multi‐factor logistic regression analysis indicate significant associations with anemia prediction in osteosarcoma patients post‐chemotherapy. Specifically, lymphocyte count (LYM) (*p* = 0.028, OR = 0.451, 95% CI = 0.221–0.918), platelet count (BLT) (*p* = 0.016, OR = 1.005, 95% CI = 1.001–1.009), serum ferritin (SF) (*p* = 0.016, OR = 0.852, 95% CI = 0.748–0.971), calcium ion (Ca) (*p* = 0.014, OR = 0.047, 95% CI = 0.004–0.536), creatinine (CREA) (*p* = 0.050, OR = 0.974, 95% CI = 0.948–1.000), D‐dimer (*p* = 0.005, OR = 1.188, 95% CI = 1.052–1.341), erythrocyte sedimentation rate (ESR) (*p* = 0.003, OR = 1.031, 95% CI = 1.010–1.052), procalcitonin (PCT) (*p* = 0.013, OR = 1.667, 95% CI = 1.114–2.493), and cancer antigen 153 (CA. 153) (*p* = 0.037, OR = 1.065, 95% CI = 1.004–1.129) were identified as significant factors. LYM, PLT, ALB, Ca, CREA, D‐dimer, ESR, PCT, and CA‐153 emerged as independent risk factors associated with anemia development post‐chemotherapy in osteosarcoma patients. These factors serve as independent predictors for the construction of the multi‐factor logistic regression model 2, as delineated in Table 3. Model 2 yielded a predictive area under the curve (AUC) of 0.872 (see Figure 4A), indicating strong discrimination ability. The calibration curve (Figure 4B) demonstrated the model's accuracy in estimating the probability of risk. Furthermore, the clinical DAC (Figure 4C) indicated the potential benefits to patients and the practical utility of this model.

**TABLE 3:** Results of multi‐factor logistic regression analysis.

Variables	Multivariate analysis		
	OR	95% CI	*p*
LYM	0.451	0.221–0.918	0.028
BLT	1.005	1.001–1.009	0.016
ALB	0.852	0.748–0.971	0.016
Ca	0.047	0.004–0.536	0.014
CREA	0.974	0.948–1.000	0.049
D‐dimer	1.188	1.052–1.341	0.005
ESR	1.031	1.010–1.052	0.003
PCT	1.667	1.114–2.493	0.013
CA‐153	1.065	1.004–1.129	0.037

#### 3.3.3 Model Building Based on Random Forest

**FIGURE 5:**
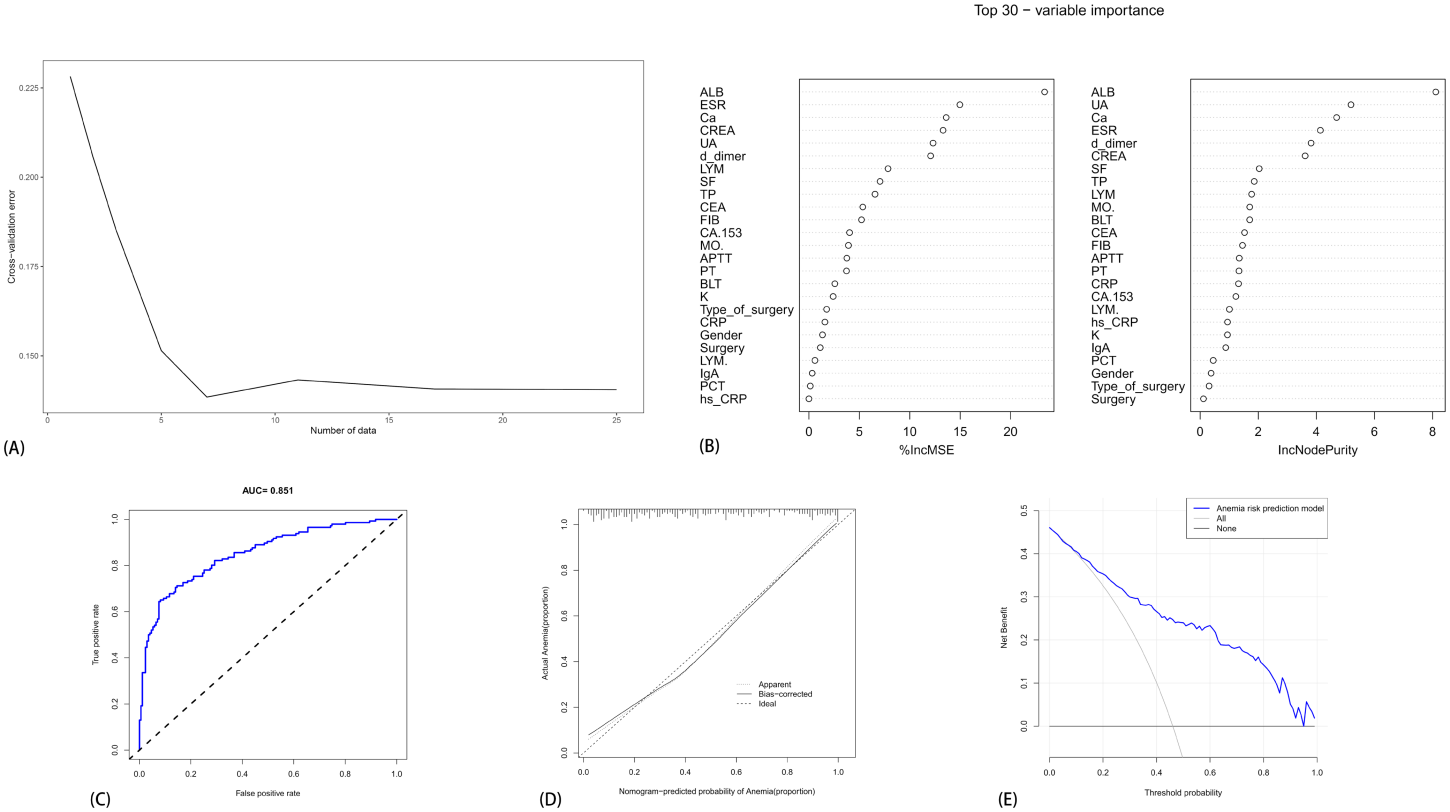
The random forest screening variables. (A) The attainment of the optimal regression effect through 10‐fold cross‐validation, retaining the seven most significant factors. (B) The calculation of the 25 most important factors utilizing two random forest algorithms, “%IncMSE” and “IncNodePurity”. (C, D, E) The ROC curve, calibration curve, and clinical decision analysis curve, respectively, based on the random forest model. (Comprising a total of 317 patients (130 females and 187 males) aged between 10 and 73 years.)

The construction of the random forest model utilized the clinical information and laboratory test results from the training set. Using a controlled method, the number of random trees (*N*trees) was initially fixed, followed by determining the optimal feature selection (mtry). Given the criterion of statistical significance (*p* < 0.05), an abundance of statistically significant factors emerged from the univariate analysis, potentially leading to model overfitting upon inclusion in the random forest model. Hence, this study selectively integrated meaningful indicators with *p* < 0.05 from general statistical analysis into the random forest model. The assignment method is delineated in Table 2, and the model's misjudgment rate under various feature selections (mtry) was computed. Notably, when mtry = 7, the misjudgment rate minimized, thus setting the value of mtry to 7, and achieving stable error tendencies, as depicted in Figure 5A. Subsequently, the random forest model ranked the importance measures of clinical information and laboratory test results, identifying seven significant risk predictors (ALB, UA, Ca, ESR, D‐Dimer, CREA, SF), as illustrated in Figure 5B. These significant independent risk factors were leveraged to construct a predictive model for anemia post‐chemotherapy in osteosarcoma, denoted as Model 3, yielding an ROC curve value of 0.851. This signifies the model's excellence and its capability to attain favorable prediction outcomes (Figure 5; Table 4).

**TABLE 4:** Final selection of random forest regression.

	%IncMSE	IncNodePurity
ALB	23.37383061	8.1104575
UA	12.31334211	5.1921788
Ca	13.61331892	4.6983698
ESR	14.97210378	4.1399627
D‐Dimer	12.07115882	3.8188321
CREA	13.30832797	3.6150188
SF	7.051761469	2.0354149

#### 3.3.4 Construction of Anemia Prediction Model Based on SVM

**FIGURE 6:**
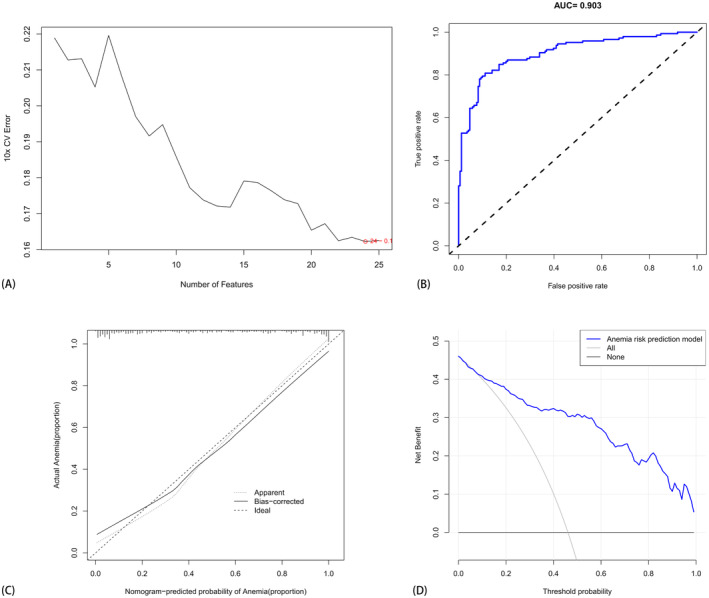
The SVM screening variables. (A) The selection of these 25 factors as the diagnostic model following SVM‐RFE calculation. (B) The ROC curve based on the SVM model, whereas (C) depicts the calibration curve, and (D) showcases the clinical decision analysis curve based on the SVM model. (Comprising a total of 317 patients (130 females and 187 males) aged between 10 and 73 years.)

The independent risk factors obtained after *t*‐test were imported into the SVM machine learning framework to construct a predictive model for anemia post‐osteosarcoma chemotherapy. Ten‐fold cross‐validation was conducted on the training set. As evidenced by Figure 6, after SVM‐RFE calculation, selecting 25 factors for the diagnostic model yielded the lowest error rate, with all included factors contributing meaningfully to diagnosis. Table 5 presents the order of importance of the 25 factors identified through SVM‐RFE. The AUC of the ROC curve obtained was 0.903, affirming the model's excellence and its capacity to achieve desirable prediction outcomes (Figure 6).

**TABLE 5:** Final selection of support vector machine (SVM).

FeatureName	AvgRank
ALB	1.1
BLT	4.4
PCT	4.6
APTT	5.2
LYM	5.5
Ca	6.3
D‐dimer	6.8
CREA	6.9
Gender	8.7
CA‐153	11.2
LYM%	11.8
ESR	13.2
CEA	15.3
TP	16
MO%	16.3
Hs‐CRP	17.1
Type of surgery	17.4
FIB	17.5
Surgery	17.8
CRP	18.2
PT	19
UA	19.3
IgA	21
SF	22.2
K	22.2

#### 3.3.5 Construction of Anemia Prediction Model Based on LASSO

**FIGURE 7:**
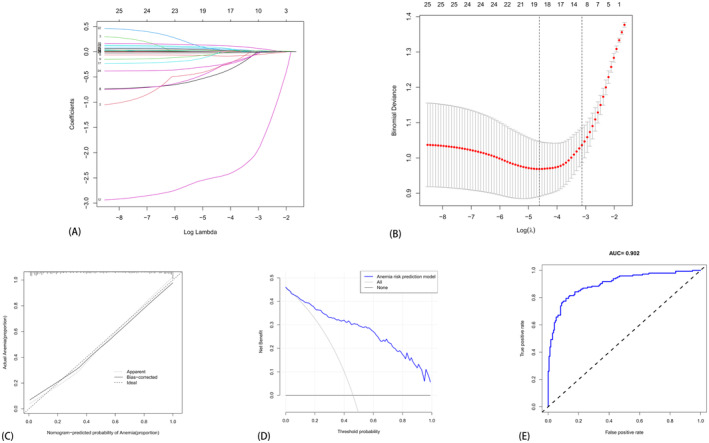
The LASSO machine learning method. (A) The LASSO regression coefficient graphs with different penalty parameter values and cross‐validation graphs of penalty terms. Each curve represents the trajectory of a coefficient of an independent variable. The ordinate represents the value of the corresponding coefficient of the independent variable, whereas the lower abscissa denotes log (*λ*), and the upper abscissa indicates the number of variables with non‐zero coefficients in the model at that time. The two dotted lines represent lambda. min and lambda. lse (left and right). (B) The results of the LASSO regression analysis of dependent variables, highlighting 19 factors exhibiting significant differences between patients with and without anemia. (C, D, E) The ROC curve, calibration curve, and clinical decision analysis curve based on the LASSO model, respectively. (Comprising a total of 317 patients (130 females and 187 males) aged between 10 and 73 years.)

The LASSO regression model was established by screening 25 meaningful variables from the *t*‐test results. Nineteen indicators relevant to the anemia prediction model for patients with osteosarcoma after chemotherapy were identified, including Gender, Surgery, LYM, MO, LYM%, BLT, TP, ALB, CA, CREA, APTT, FIB, D‐dimer, CRP, ESR, PCT, IgA, CEA, and CA.153. A predictive model was constructed accordingly. Refer to Figure 7A,B for details. The AUC of the ROC curve was calculated as 0.902, confirming the model's excellence and its ability to achieve favorable prediction outcomes, as illustrated in Figure 7C,D.

#### 3.3.6 Comparison Between the above Models

Comparing several machine learning models, the AUC of the logistic regression (LR) prediction model is 0.895 in the single‐factor scenario, whereas the LR multi‐factor AUC is 0.872. The RF prediction model yielded an AUC of 0.882 (95% CI: 0.808–0.892, *p* < 0.05). The SVM prediction model achieved an AUC of 0.903 (95% CI: 0.869–0.937, *p* < 0.05), whereas the AUC of the LASSO prediction model is 0.902 (95% CI: 0.868–0.936, *p* < 0.05). In the training set, the predictive performance of each model for anemia after osteosarcoma chemotherapy was ranked as follows: SVM > LASSO > single‐factor logistic regression > multi‐factor logistic regression > RF.

#### 3.3.7 Model Construction After Intersection

**FIGURE 8:**
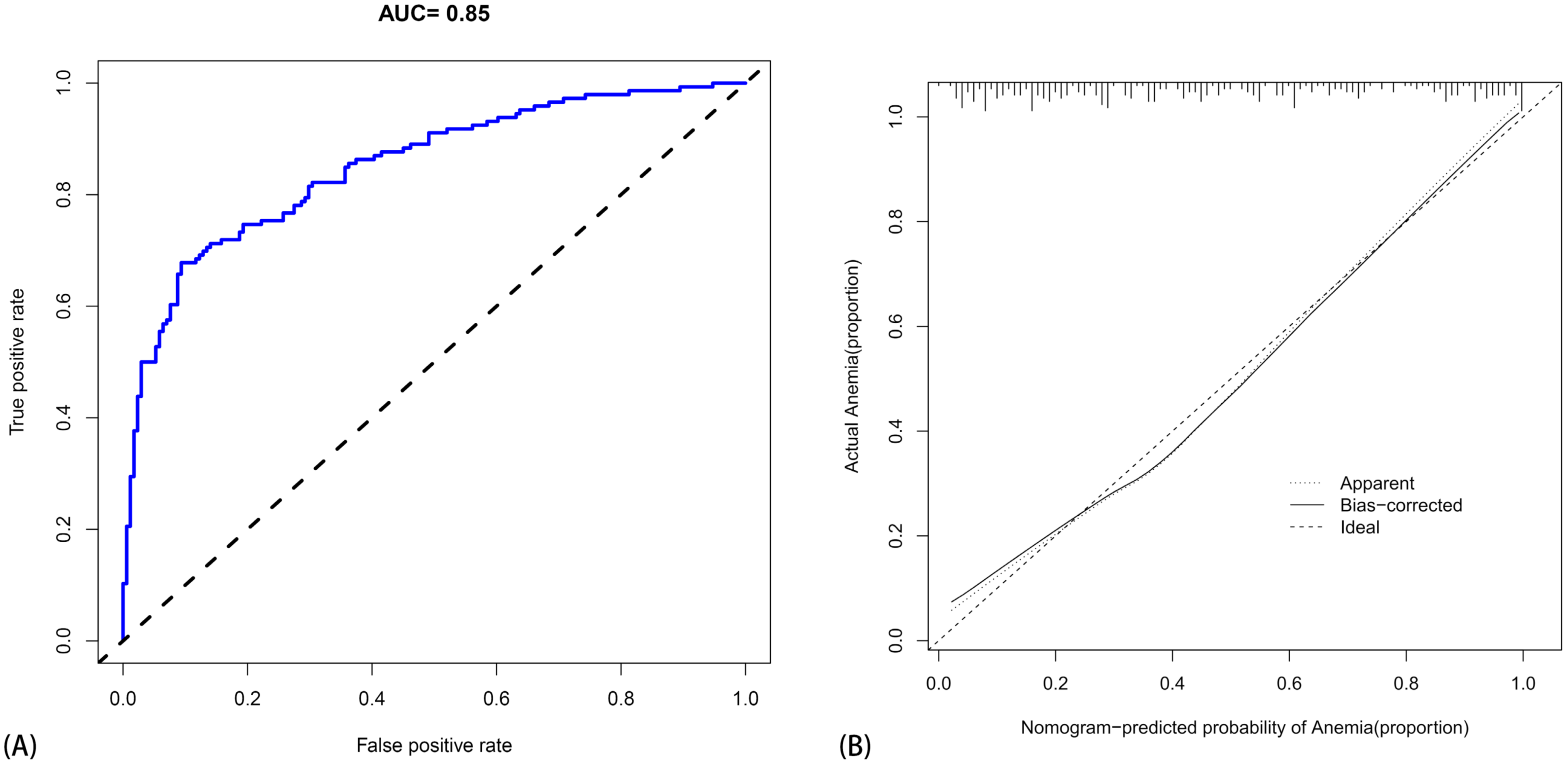
The ROC curve for predicting anemia after the intersection, depicted in (A), along with the Calibration curve for predicting anemia after the intersection shown in (B). (Comprising a total of 317 patients (130 females and 187 males) aged between 10 and 73 years.)

Common risk factors such as ALB, Ca, CREA, D‐dimer, and ESR were identified through the analysis of each risk prediction model, indicating their significant impact on anemia. However, selecting too many risk factors may reduce the efficiency of the machine learning model, while too few may lead to overfitting. Thus, to optimize the risk factors and achieve an ideal prediction model, we combined the risk factors from the aforementioned models to construct a new model (Model 6) with an AUC of 0.85. Compared with previous models, the difference in Model 6 was not statistically significant, suggesting its potential to achieve ideal prediction results (Figure 8A,B). Simplifying the model index can aid in reducing the necessary data collected by clinicians and effectively reduce their workload.

### 3.4 Construction of Anemia Prediction Model

**FIGURE 9:**
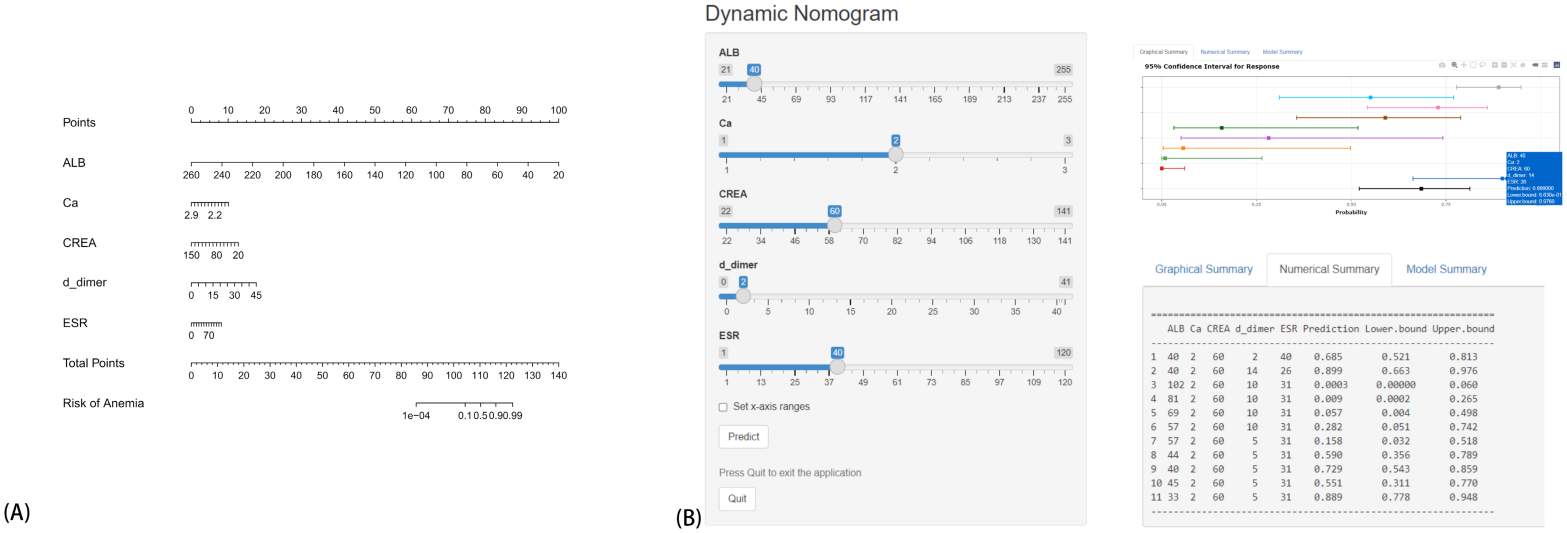
(A) Nomogram illustrating a novel model for predicting the risk of anemia following chemotherapy for osteosarcoma. (B) Individualized risk prediction using the online nomogram. (Comprising a total of 317 patients (130 females and 187 males) aged between 10 and 73 years.)

To forecast the individualized risk of anemia in each osteosarcoma patient after chemotherapy, five variables were identified based on the intersection of the training set model. A column graph was generated according to the results of multivariate Logistic regression model analysis. The cumulative score values of each variable were summed, yielding a total score that could predict the incidence of anemia in osteosarcoma patients after chemotherapy, as illustrated in Figure 9A. Additionally, an online nomogram was developed to facilitate clinicians' application (https://anemic‐prediction‐of‐osteosarcoma.shinyapps.io/DynNomapp/). By inputting the specific values of each variable of the five indicators, clinicians can easily determine the individualized probability of anemia occurrence, as demonstrated in Figure 9B.

### 3.5 Verification Set Model Validation

**FIGURE 10:**
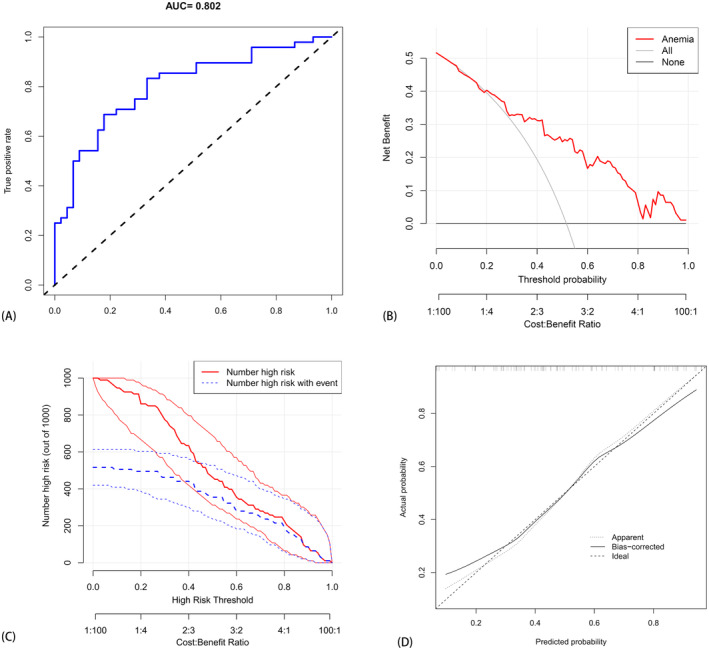
The validation of the new model using the validation set. (A) The AUC curve of the model applied to the verification set. (B) The clinical decision analysis curve of the model applied to the validation set, whereas (C) depicts the clinical impact curve of the model applied to the validation set. Finally, (D) exhibits the calibration curve of the model applied to the verification set.

The risk prediction model for anemia after chemotherapy in osteosarcoma was assessed on a validation set comprising 93 cases, with 48 cases exhibiting anemia post‐chemotherapy and 45 cases being non‐anemic. The AUC of the prediction model in the validation set was 0.802 (95% CI, 0.713–0.891), with the calibration curve indicating good agreement between the prediction model and the actual probability. DAC and CIC demonstrate that the model constructed after intersection application possesses predictive and application value. The DCA curve, based on this foundation, indicates a significant net benefit across various threshold probabilities, particularly in the range of 30%–80%. However, for threshold probabilities less than 30%, DCA exhibits a net benefit comparable to predicted positive results for all patients. Similarly, CIC assessed the clinical benefit of the model, revealing predictive and application value in the column chart. Therefore, we assert that the model fits well in both the training set and the verification set, with accurate prediction capabilities (Figure 10).

## 4 Discussion

The model developed in this study presents an excellent predictive tool for individualized anemia risk assessment in osteosarcoma patients following chemotherapy. Although prior research has explored the application of machine learning in prognostic risk prediction for osteosarcoma patients, there is a dearth of literature addressing machine learning's role in predicting anemia risk post‐chemotherapy. Thus, we endeavored to investigate this area. Our findings identified 25 indicators, including Gender, Surgery, Type of surgery, LYM%, MO%, LYM, BLT, TP, ALB, SF, K, CA, CREA, UA, PT, APTT, FIB, D‐dimer, CRP, hs‐CRP, ESR, PCT, IgA, CEA, and CA‐153, as independent risk factors for anemia. Through univariate and multifactor logistic regression analyses, as well as the integration of RF, SVM, and LASSO methods, five indices—ALB, Ca, CREA, D‐dimer, and ESR—were identified as significant variables. The resultant model exhibited high predictive capability.

Albumin (ALB) is a pivotal protein in human plasma, crucial for maintaining blood volume and fluid balance. Diminished plasma albumin concentrations can reduce plasma colloid osmotic pressure, potentially leading to interstitial fluid accumulation and edema, thereby impacting the patient's microenvironment [18]. Our study revealed a significant negative association between albumin levels and anemia development following chemotherapy in osteosarcoma patients, consistent across all models. We hypothesize two primary mechanisms contributing to anemia. First, osteosarcoma, as a wasting disease, may escalate albumin consumption, leading to reduced levels of albumin and subsequent malnutrition, thereby affecting the erythropoietic environment and increasing susceptibility to anemia. Second, diminished serum albumin levels may result in inadequate erythropoietin production. Erythropoietin, primarily synthesized by peritubular cells in the kidneys, regulates erythroprogenitor cell proliferation and differentiation in the bone marrow. Decreased peritubular cell function leads to reduced circulating erythropoietin levels, exacerbating anemia [19]. Previous studies have demonstrated a significant correlation between serum albumin levels, renal function, and anemia in chronic kidney disease patients, aligning with our research findings [20]. Hence, we posit a close association between albumin levels and anemia occurrence.

Calcium ion plays a vital role in cellular function regulation, primarily through maintaining the concentration gradient of Ca^2+^ ions across the cell membrane. Alterations in this concentration gradient can detrimentally affect cell function, potentially leading to cellular dysfunction or even cell death. The regulation of Ca^2+^ concentration relies on calcium channels present in the cell membrane, which help maintain the concentration gradient between the intracellular and extracellular environments, with extracellular Ca^2+^ concentrations typically being higher than intracellular levels [21]. Our investigation revealed a modest negative correlation between calcium ion concentration and anemia, consistent with previous research. We hypothesize that post‐chemotherapy, some osteosarcoma patients may experience hypocalcemia, leading to an increase in calcium ion concentration within red blood cells relative to the extracellular environment. This disparity may result in heightened permeability and fragility of red blood cell membranes, leading to red blood cell destruction and subsequent anemia [22]. Additionally, chemotherapy‐induced apoptosis of hematopoietic stem cells in osteosarcoma patients may be promoted by factors such as IFN‐r and TNF‐α, triggering biochemical reactions in target cells, including elevated calcium ion levels, DNA degradation, and apoptosis [23]. The apoptosis of hematopoietic stem cells results in a direct decrement in patients' hematopoietic function, thereby triggering anemia.

The kidneys play a crucial role in regulating water and electrolyte balance, maintaining acid–base equilibrium, and exhibiting endocrine functions such as renin and erythropoietin production [24]. In our investigation, we observed a weak positive correlation between anemia severity and elevated serum creatinine levels. This finding suggests that osteosarcoma patients undergoing chemotherapy may experience immunosuppression, disrupting normal kidney metabolism and hematopoietic function. Increased serum creatinine levels often indicate renal injury, which may be associated with chemotherapy drug accumulation [25]. Renal injury can reduce endogenous erythropoietin secretion, leading to decreased red blood cell lifespan and impaired bone marrow hematopoietic function, ultimately contributing to anemia. Furthermore, kidney function impairment can hinder bone iron absorption, disrupt the hematopoietic microenvironment, and elevate anemia risk.

D‐dimer serves as a degradation product of crosslinked fibrin, reflecting the activation status of both coagulation and fibrinolysis systems within the body [26]. Elevated levels of D‐dimer signify concurrent activation of these systems, often indicative of a pre‐thrombotic or hypercoagulable state [27]. Our study observed a positive correlation between increased D‐dimer levels and heightened incidence of anemia in osteosarcoma patients post‐chemotherapy, aligning with prior research. Malignant tumors, including osteosarcoma, can robustly accelerate plasma coagulation, primarily through direct activation of prothrombin to thrombin by tumor cells. Additionally, the rapid growth of osteosarcoma can lead to tissue infiltration and damage to surrounding blood vessels, disrupting the delicate balance between the anticoagulant and coagulation systems, consequently elevating D‐dimer levels and predisposing individuals to coagulation disorders, bleeding tendencies, and anemia [28]. Chemotherapy in osteosarcoma patients may exacerbate this mechanism, further increasing D‐dimer levels.

Erythrocyte sedimentation rate (ESR) denotes the rate at which erythrocytes settle under specific conditions, influenced by changes in plasma composition or red blood cell characteristics. In cases of severe anemia, reduced red blood cell surface area diminishes plasma resistance, hastening sedimentation rates [29]. Cancer, closely intertwined with inflammation, often manifests elevated ESR, serving as a marker for inflammatory and oncological conditions [30]. The inflammatory response within osteosarcoma lesions impedes tumor cell apoptosis, fosters tumor cell proliferation, and inhibits immune factor production. Following chemotherapy, compromised immune function in osteosarcoma patients prompts varying degrees of inflammatory factor release, accelerating ESR. Heightened inflammatory cytokines induce ferrimodulin elevation, impeding gut iron absorption and prompting ferritin elevation as an acute phase reactant, contributing to anemia [31]. Conversely, cancer progression correlates with accelerated ESR, fostering favorable conditions for cancer cell aggregation, adhesion, and metastasis, exacerbating osteosarcoma development and consequent anemia occurrence. Hence, accelerated ESR effectively reflects post‐chemotherapy anemia in osteosarcoma patients.

Cancer‐related anemia (CRA) denotes a decline in red blood cell count or hemoglobin levels attributed to the tumor itself or ensuing radiotherapy and chemotherapy [32]. It is a prevalent complication among cancer patients and may be linked to the tumor microenvironment. The tumor microenvironment encompasses surrounding fibroblasts, endothelial cells, and various immune cells, all of which play a crucial role in cancer development [33]. This complex microenvironment not only influences the proliferation, migration, and invasion of tumor cells but also may contribute to the onset of cancer‐associated anemia. Approximately 50% of tumor patients exhibit anemia symptoms, with the incidence soaring to 90% among those with advanced tumors or undergoing radiotherapy and chemotherapy. Osteosarcoma patients similarly experience anemia complications post‐chemotherapy. We developed anemia risk prediction models utilizing ALB, Ca, CREA, D‐dimer, and ESR. Online charts were generated to illustrate these models. Within the chart, each indicator is assigned a specific value based on predetermined ratios. By inputting individual information and corresponding values, the total score can be computed, facilitating the prediction of anemia risk. Specifically, for an osteosarcoma patient experiencing post‐chemotherapy anemia, relevant points can be located in the graph based on the patient's information. The summation of all points yields the total score, which can then be correlated with the probability of the predicted event.

Individualized treatment of osteosarcoma patients is crucial for prognosis. Anemia, a common complication following chemotherapy, can adversely affect prognosis. Hence, early and accurate diagnosis or prediction of anemia, coupled with timely intervention, can significantly enhance patient outcomes. The nomogram developed in our study exhibits considerable promise for clinical application. Clinicians can utilize this model to assess the risk of post‐chemotherapy anemia in osteosarcoma patients, thereby offering personalized diagnosis and treatment guidance, ultimately improving patient prognosis.

This study boasts several strengths. First, it pioneers the use of common clinical indicators to develop a predictive model for anemia risk following osteosarcoma chemotherapy. The inclusion of a wide array of 100 clinical data indicators lends our findings significant research and practical value. Second, the five variables featured in the chart are routine laboratory test indicators readily accessible in most medical settings, thus ensuring broad applicability of the model. Lastly, the nomogram represents a visually intuitive predictive tool that can be seamlessly integrated into clinical practice. It empowers healthcare professionals to swiftly diagnose and evaluate a patient's condition through simple calculations, aiding in the classification and severity assessment of anemia and facilitating the formulation of individualized treatment plans. However, our study has several limitations that warrant consideration. First, the involvement of only three institutions restricts the breadth of evidence‐based medical data, highlighting the need for further validation through prospective, multicenter studies. Second, although these five measures play a crucial role in anemia risk modeling, their lack of specificity for osteosarcoma limits their clinical utility. Nonetheless, given the absence of specific serum markers for osteosarcoma, anomalies in nonspecific markers may still contribute to supporting the diagnosis of post‐chemotherapy anemia. Moreover, akin to other retrospective studies, our research is susceptible to selection bias. Furthermore, different subtypes of osteosarcoma may influence oncologic anemia, but the number of cases of other osteosarcoma subtypes is very limited worldwide. Thus, further studies investigating the association between osteosarcoma subtypes and anemia may require accumulating larger datasets in the future. Nevertheless, despite these constraints, our retrospective analysis retains clinical relevance and offers valuable insights for guiding future prospective investigations.

## 5 Conclusion

Our study has successfully developed and validated a novel model for predicting the occurrence of anemia following chemotherapy in osteosarcoma patients, integrating ALB, Ca, CREA, D‐dimer, and ESR as predictive variables. DCA and CIC underscored the clinical significance and substantial net benefit of the nomogram. Moving forward, prospective investigations are warranted to further expand the clinical applicability and robustness of our model.

## Author Contributions

**Zhiping Su:** conceptualization (equal), data curation (equal), writing – original draft (equal), writing – review and editing (equal). **Zhiwei Nong:** writing – original draft (equal), writing – review and editing (equal). **Feihong Huang:** data curation (equal). **Chengxing Zhou:** data curation (equal). **Chaojie Yu:** conceptualization (equal), writing – original draft (equal), writing – review and editing (equal).

## Ethics Statement

The study was carried out in accordance with the Declaration of Helsinki statement. The present study was approved by the Ethics Committee of Guangxi Medical University Cancer Hospital. The need for written informed consent was waived by Guangxi Medical University Cancer Hospital Ethical Review Committee due to the retrospective nature of the study. Written informed consent was not obtained from the individual(s) for the publication of any potentially identifiable images or data included in this article.

## Consent

The authors have nothing to report.

## Conflicts of Interest

All authors declare that the research was conducted in the absence of any commercial or financial relationships that could be construed as a potential conflict of interest.

## Data Availability

All data that support the findings of this study are included in this manuscript and its supplementary information files. Further enquiries can be directed to the corresponding author.
